# Effectiveness and safety of once-weekly semaglutide: findings from the SEMACOL-REAL retrospective multicentric observational study in Colombia

**DOI:** 10.3389/fendo.2024.1372992

**Published:** 2024-06-25

**Authors:** Daisy C. Buenaventura-Collazos, Andrés F. García-Ramos, Carlos M. Balcázar-Valencia, Carolina Aguilar-Londoño, Nicolás Coronel-Restrepo, Claudia Y. Monsalve-Arango, Diana P. Cuesta-Castro, Alex Ramírez-Rincón

**Affiliations:** ^1^ Endocrinology Department, Universidad Pontificia Bolivariana, Medellín, Colombia; ^2^ Endocrinology Department, Universidad del Valle, Cali, Colombia; ^3^ Endocrinology Department, Universidad Libre, Cali, Colombia; ^4^ Endocrinology Department, Clínica Las Américas – Aúna, Medellín, Colombia; ^5^ Endocrinology Department, Clínica Medellín – Quirón Salud, Medellín, Colombia

**Keywords:** diabetes mellitus, glucagon-like peptides, hypoglycemic agents, adults, Colombia

## Abstract

**Introduction:**

Diabetes stands as one of the leading causes of death worldwide. Glucagon-like peptide-1 receptor agonists rank among the most effective medications for lowering blood glucose and body weight, as well as reducing cardiovascular risk in individuals with diabetes. Observational studies complement experimental evidence in new settings, different populations, and real-world healthcare practices.

**Methods:**

A multicentric observational study of adults with type 2 diabetes treated with once-weekly subcutaneous semaglutide in four health centers in Colombia was conducted. The protocol for the present study was not pre-registered.

**Results:**

Data from 186 patients were included. Most patients were women (57%) with a mean age of 62.8 ± 12.1 years. One year of once-weekly semaglutide usage was associated with a mean reduction in HbA1C of −1.47% (95% CI −1.76, −1.17), weight loss of −4.23 kg (95% CI −5.34, −3.12), and albumin/creatinine ratio of −18.6 mg/g (95% CI −60.2, −5.9). Approximately half the treated patients achieved a level of HbA1c ≤7% by the end of follow-up. Adverse events were rare and consistent with clinical trial safety profiles.

**Conclusion:**

In Colombia, administering semaglutide subcutaneously once a week over a 1-year period led to an average weight loss of 4.2 kg and a decrease of 1.4% in HbA1c.

## Introduction

Five conditions account for 80% of deaths related to chronic non-communicable diseases, with diabetes mellitus (DM) being one of the leading contributors (1). According to the International Diabetes Federation’s 2021 estimates, 537 million adults worldwide were living with diabetes, and 90% of them had type 2 diabetes (T2D) (2). In Latin America, approximately 10% of adults live with diabetes, with Colombia having the second-largest population of individuals with T2D (3).

A treatment strategy based on stricter glucose control, intended to reduce glycated hemoglobin (HbA1c) levels to close to those of a person without diabetes, has been shown to reduce mortality and the appearance of micro- and macrovascular complications in people with DM (4–6). However, only approximately half of those undergoing treatment reached the target HbA1c level of less than 7% (7, 8).

The landscape of T2D treatment has evolved, and current recommendations emphasize not only glycemic control but also reducing the risk of conditions such as cardiovascular disease and kidney disease, aiding weight loss in people with obesity, and promoting a patient-centered approach (9, 10).

The once-weekly subcutaneous glucagon-like peptide-1 receptor agonist (GLP1-RA), semaglutide (OWS), has shown superior efficacy in glycemic control and weight loss compared to placebo and multiple comparators, as demonstrated by the SUSTAIN clinical trial program (11). Notably, OWS also exhibited cardiovascular protective effects in individuals at high risk for diabetes-related cardiovascular issues, and there is promising evidence suggesting its kidney-protective effects (12). OWS was first introduced in Colombia in 2020 with total healthcare coverage as an adjunct to diet, exercise, and other hypoglycemic medications to improve glycemic control and reduce the risk of major adverse cardiovascular events.

The SURE program complemented the data from the SUSTAIN clinical trials as it investigated OWS in a real-world setting in several European countries (13–20) and Canada (21). Real-world studies provide valuable insights into the effects of medication across a diverse population, encompassing a broader age range and different socio‐economic settings. These studies are particularly beneficial in assessing the impact of medication on individuals with different healthcare insurance policies, people with other comorbidities usually excluded from clinical trials, and different medication combinations for treating T2D.

Diabetes treatment in Latin American countries differs significantly from that provided in Europe and North America, a distinction influenced by cultural, social, economic, and genetic factors, as well as healthcare coverage. Colombia, with its universal public healthcare system, initiated full reimbursement of OWS in 2020. Our aim was to assess the 1-year effect of OWS in routine clinical practice across multiple healthcare centers in Colombia.

## Methods

### Study design

The SEMACOL-REAL study was a multicentric observational investigation using electronic medical records (EMR). The study designated the date of first OWS prescription as the index date for analysis. Data from prescriptions issued between June 2020 to July 2022 were collected. Follow-up data encompassed information from the index date and 3-, 6-, and 12-month follow-ups.

The protocol for the present study was not pre-registered.

### Study population

The study included adult patients (≥18 years) with T2D and a prescription for OWS from four medical centers in two cities in Colombia. EMRs from patients with less than one follow-up visit or those with no registered medication use were excluded.

### Endpoints

The study’s primary endpoint was changed from baseline to 1-year follow-up in HbA1c (%). Secondary endpoints were divided into the following categories: Glucose control, body weight, kidney endpoints, diabetes treatment, and other clinically meaningful measurements. Glucose control endpoints included changes in HbA1c (%) and fasting glucose (mg/dL) on each visit. Body weight outcomes included changes in body mass index (BMI) (kg/m²) and body weight (kg). Kidney endpoints encompassed the change in urine albumin-to-creatinine ratio (ACR) (mg/g) and estimated glomerular filtration rate (eGFR) calculated using the CKD-EPI formula. The endpoints for diabetes treatment included changes in antidiabetic medication usage and insulin dosage. Other clinically meaningful measurements included differences in blood pressure (mmHg) and blood lipids. Safety endpoints included acute myocardial infarction events, stroke, and gastrointestinal adverse events recorded on each visit. Only partial data for the endpoints were available from one of the institutions, which closed permanently during the study period.

### Statistical analyses

Continuous variables in the data distribution are expressed as mean and standard deviation (SD) or median and interquartile range (IQR). Normal distribution was evaluated using the Kolmogorov–Smirnov test. Qualitative variables are presented as absolute frequencies and proportions. Changes in the continuous variables over time of the main outcomes were analyzed using a linear mixed-effects model with a random slope for each participant, adjusted for baseline value, age, and sex, and to calculate *p*-values and confidence intervals, the *m-l-1* heuristic was used. As a sensitivity analysis, a model was specified using the center as a covariate, and for changes in body weight and BMI, a model incorporating the previous use of GLP1-RA was also computed. Secondary outcomes were analyzed using an unadjusted linear mixed-effects model. No adjustments were made for multiple comparisons. All analyses were performed using R Statistical Software (V4.3.1; R Core Team 2023) and RStudio (V RStudio).

### Ethical considerations

The ethical considerations of this protocol adhere to the amendment made in 2013 during the general assembly of the World Medical Association in Fortaleza, Brazil; the Declaration of Helsinki; the Ethical Principles for Medical Research Involving Human Subjects; and the Fourth Version of the Ethical Guidelines for Health-Related Research with Human Subjects prepared by the Council for International Organizations of Medical Sciences (CIOMS) in collaboration with the World Health Organization (WHO). The ethical considerations of this protocol align with the Colombian Ministry of Health’s Resolution 8430. The study protocol received approval from Clínica Bolivariana, Clínica Las Américas – AUNA, and Clínica Medellín – Quirón Salud institutional review board (IRB). Clínica Comfenalco Calí used an external IRB. The approval letters and the IRB codes and contacts are provided in the **Supplementary Material**.

## Results

**Figure 1:**
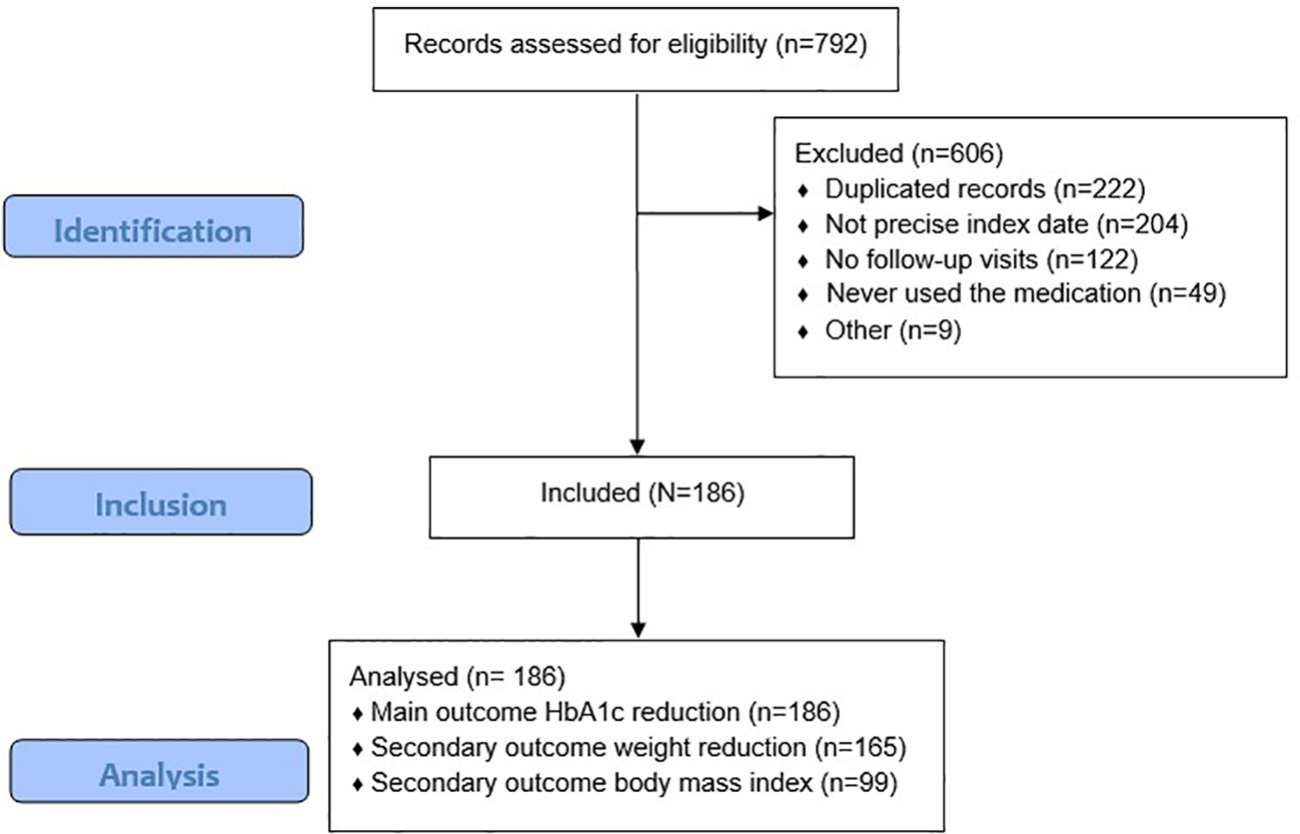
Patients flow diagram. *Number of patients per-center: Clínica Comfenalco Cali: 79, Clínica Medellín: 53, Clínica Universitaria Bolivariana: 50, Clínica Las Américas AUNA: 4.

Data from a total of 792 EMRs of patients initiating OWS were reviewed. Following the exclusion criteria for duplications, imprecise index dates, lack of registered medication use, and absence of follow-up visits, 606 records were excluded. Patient disposition is shown in **Figure 1**.

Baseline characteristics are summarized in **Table 1**. At baseline, 57% of the patients were women, with a mean age of 62.8 (SD 12.1) years. The initial HbA1c was 8.4% (IQR 7.2–9.9), ACR was 30.3 mg/g (IQR 8–157), and GFR was 77.5 (SD 23.9). Most patients (69%) used basal insulin, 40% switched from another GLP1-RA to OWS, and 28% used at least one daily dose of prandial insulin. Data completeness for some variables was not universal. **Table 1** presents the number of subjects with available data.

**Table 1:** Baseline patient’s characteristics.

		*n*
**Age, years, mean (SD)**	62.8 (12.1)	186
**Female, *n* (%)**	105 (57)	186
**Weight, kg, median (IQR)**	84.5 (71.7–97.5)	186
**BMI, kg/m², mean (SD)**	31.2 (5.74)	99
**Fasting serum glucose, mg/dL, median (IQR)**	152 (120–203)	186
**HbA1c, %, median (IQR)**	8.4 (7.2–9.9)	186
**Systolic blood pressure, mmHg, mean (SD)**	129.3 (16.8)	102
**Diastolic blood pressure, mmHg, mean (SD)**	75.5 (10.9)	102
**Total cholesterol, mg/dL, mean (SD)**	147.8 (37.8)	90
**LDL cholesterol, mg/dL, mean (SD)**	72.4 (37.8)	86
**HDL cholesterol, mg/dL, mean (SD)**	40.8 (10)	87
**Triglycerides, mg/dL, mean (SD)**	181.6 (81.8)	89
**eGFR, mL/min/1.73 m², mean (SD)**	77.5 (23.9)	99
**Myocardial infarction, *n* (%)**	43 (40.2)	107
**Stroke, *n* (%)**	3 (2.8)	107

BMI, body mass index; SD, standard deviation; IQR, interquartile range; LDL, low-density lipoprotein; HDL, high-density lipoprotein. Differences in the number of patients for each variable is due to missing data related to the retrospective design and the source (EMR) of the data.

### Glucose control

**Figure 2:**
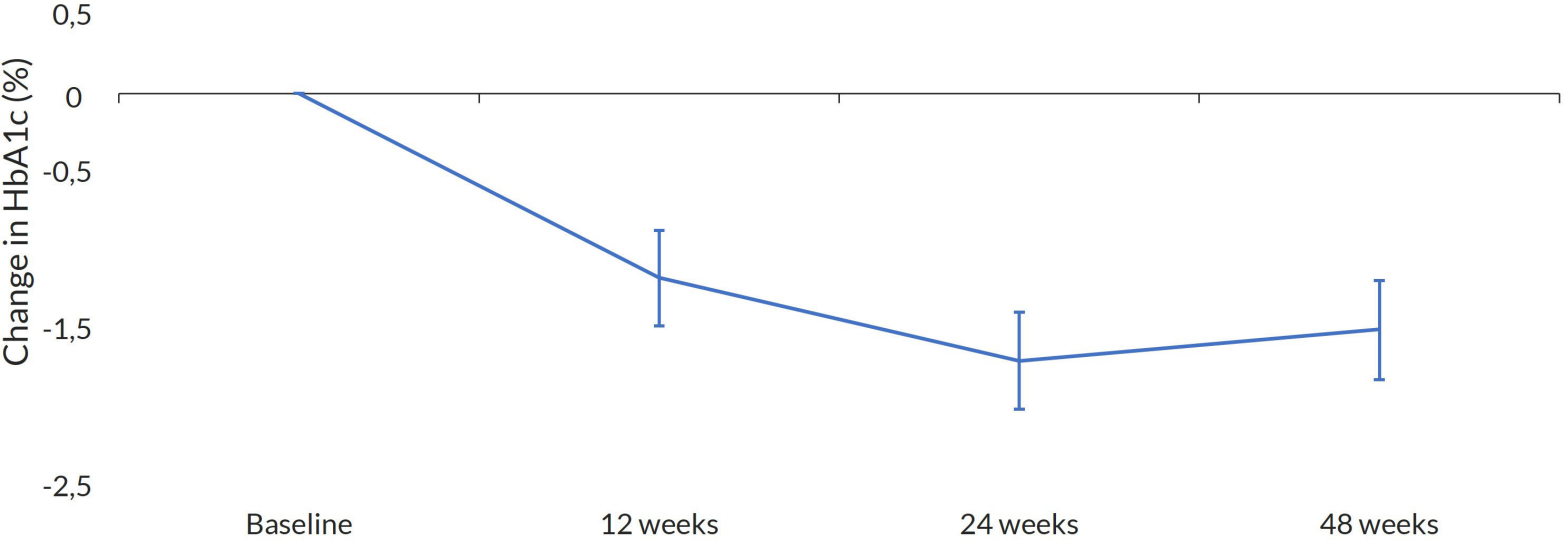
Change in HbA1c. Mean change at every visit with 95%CI.

**Figure 3:**
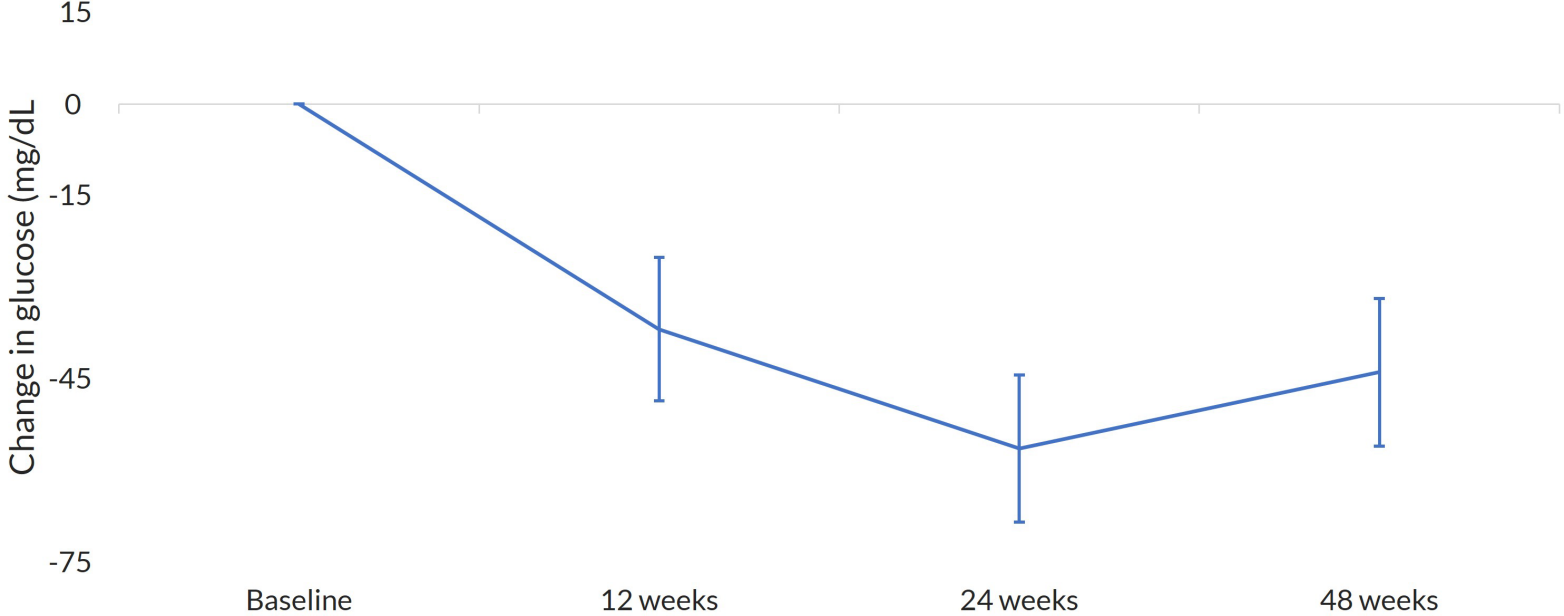
Change in glucose. Mean change at every visit with 95%CI.

At the end of the study (EOS), OWS significantly reduced HbA1c and fasting glucose levels. The median decrease of HbA1c was −1.47% (95% CI −1.76, −1.17; *p* < 0.001), and for fasting glucose, it was −42.18 mg/dL (95% CI −56.96, −27.40; *p* < 0.001). A reduction over time for both HbA1c and glucose was observed (**Figures 2**, **3**). At baseline, only 24.3% of the patients had an HbA1c < 7%; at EOS, the proportion of patients with reasonable glycemic control increased to 47%. For older patients, those over 65 years, a glycemic target of HbA1% below 8% was used. The proportion of patients meeting this target increased from 56% at baseline to 80% at EOS.

### Body weight

**Figure 4:**
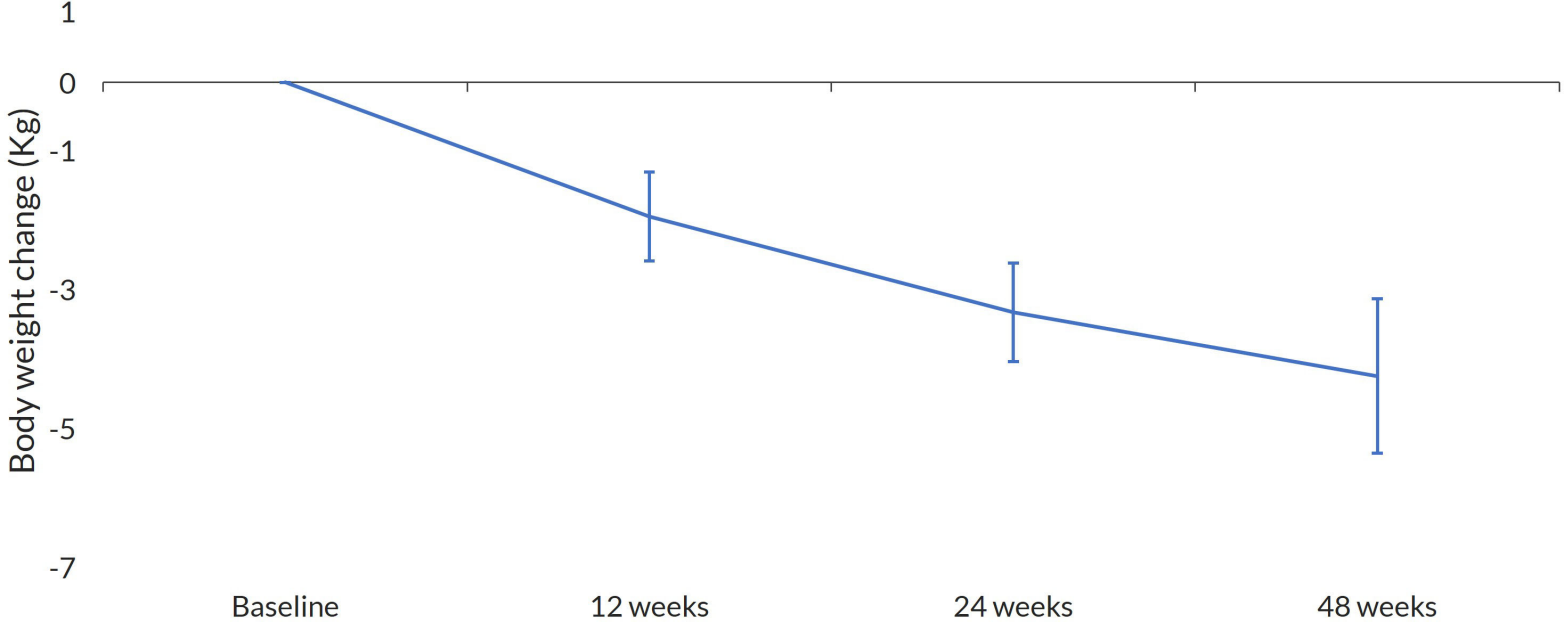
Change in body weight. Mean change at every visit with 95%CI.

The mean weight reduction was −4.23 kg (95% CI −5.34, −3.12; *p* < 0.001), as illustrated in **Figure 4**. A decrease in weight over time was also observed. When analyzed by sex, women exhibited a more significant weight reduction between visits of 9.7% (95% CI −11.7, −0.22; *p* < 0.001), as did patients aged 65 and older, with a mean weight reduction between visits of 11.6% (95% CI −19.0, −4.0; *p* < 0.001). When combined, older women had the most significant weight reduction between visits of 15.3% (95% CI −30.0, −3.0; *p* < 0.001). The use of OWS also significantly reduced BMI by −3.14 kg/m² (95% CI −4.24, −2.03; *p* < 0.001). Changes in body weight and BMI showed no major differences in the model adjusted for previous use of other GLP1-RA (**Supplementary Material**).

### Kidney endpoints

ACR diminished significantly, −18.6 mg/g (95% CI −60.2, −5.9; *p* < 0.001), while GFR remained unchanged.

### Diabetes treatment endpoints

There was a decrease in the basal insulin dose, with a median reduction of −3.0 IU (95% CI −6.0, −1.5; *p* < 0.001). The proportion of patients using basal insulin decreased from 64.7% at baseline to 55.4% at EOS (*p* = 0.008). The median reduction in prandial insulin dose was 7.5 units (95% CI −18.0, 0.0; *p* = 0.058).

The most frequently used antidiabetic drug was metformin, and the most common combination was metformin with a sodium-glucose cotransporter 2 (SGLT2) inhibitor. The frequencies of antidiabetic drug use are shown in **Table 2**. All patients receiving a DPP4 inhibitor at the index date stopped using it after semaglutide prescription.

**Table 2:** Medication use.

	Index date *n* = 107	EOS *n* = 74
Monotherapy		
Metformin, *n* (%)	22 (20.5)	17 (22.9)
SLGT2 inhibitors, *n* (%)	6 (5.6)	8 (10.8)
DPP4 inhibitors, *n* (%)	2 (1.8)	0
Combination therapy		
Metformin + SLGT2 inhibitors, *n* (%)	40 (37.3)	39 (52.7)
Metformin + DPP4 inhibitors, *n* (%)	7 (6.5)	0
SLGT2 inhibitors + DPP4 inhibitors, *n* (%)	1 (0.9)	0
SLGT2 inhibitors + Sulfonylurea, *n* (%)	1 (0.9)	0
Metformin + SLGT2 inhibitors + DPP4 inhibitors, *n* (%)	9 (8.4)	0
Metformin + SLGT2 inhibitors + Sulfonylurea, *n* (%)	2 (1.8)	1 (1.3)

SLGT2, sodium glucose cotransporter 2; DPP4, Dipeptidyl Peptidase IV.

### Other clinically meaningful measurements

Blood pressure and lipid measurements did not exhibit significant variation at EOS. **Table 3** shows the mean changes between the index date and EOS.

**Table 3:** Other clinical meaningful measurement differences.

	ID, median (IQR)	EOS, median (IQR)	EOS difference vs. ID (95% CI)
**Systolic blood pressure**	130 (20)	120 (12.3)	−6.58 (−10.33, −2.83; *p*. 0.001)
**Diastolic blood pressure**	74.5 (10)	70.0 (10)	−5.12 (−8.10, −2.14; *p*. 0.001)
**Total cholesterol, mg/dL, mean (SD)**	139 (55.5)	129 (59)	−15.24 (−20.5, −1.0; *p*. 0.078)
**LDL cholesterol, mg/dL, mean (SD)**	70 (50.6)	60 (42.5)	−6.57 (−13.61, 0.048; *p*. 0.068)
**HDL cholesterol, mg/dL, mean (SD)**	39 (11.5)	40 (13.2)	−4.11 (−6.54, −1.68; *p*. 0.001)
**Triglycerides, mg/dL, mean (SD)**	166 (86)	147 (87.8)	−18.99 (−40.96, 2.99; *p*. 0.090)

SD, standard deviation; CI, confidence interval; LDL, low-density lipoprotein; HDL, high-density lipoprotein; ID, index date; EOS, end of study.

### Safety

There were no cardiovascular events reported during the study duration. There was no change in the trend of hypoglycemia events during follow-up. No constipation or bowel obstruction events were reported. The frequency of adverse events is reported in **Table 4**.

**Table 4:** Frequency of adverse events.

Adverse event	12 weeks	24 weeks	48 weeks
Hypoglycemia			
Level 1, *n* (%)	7 (3.7)	6 (3.2)	7 (3.7)
Level 2, *n* (%)	1 (0.5)	0 (0)	0 (0)
Level 3, *n* (%)	0 (0)	0 (0)	0 (0)
Gastrointestinal			
Pancreatitis, *n* (%)	0 (0)	0 (0)	0 (0)
Constipation/Bowel obstruction, *n* (%)	0 (0)	0 (0)	0 (0)
Nausea, *n* (%)	5 (2.6)	3 (1.6)	0 (0)
Vomit, *n* (%)	2 (1.0)	3 (1.6)	0 (0)
Abdominal pain, *n* (%)	3 (1.6)	2 (1.0)	3 (1.6)
Diarrhea, *n* (%)	1 (0.5)	0 (0)	1 (0.5)
Cardiovascular events			
Acute myocardial infarction, *n* (%)	0 (0)	1 (0.5)	0 (0)
Stroke, *n* (%)	0 (0)	0 (0)	0 (0)

## Discussion

OWS has recently become available in several countries in America, and this study represents the first reported real-life experience in the region. In Colombia, other GLP1-RAs (exenatide, dulaglutide, and liraglutide) were already available, and with full reimbursement by the healthcare system. At the time of the cohort’s index, more than one-third of the patients were already on GLP1-RA therapy before switching to OWS. While the motivations for the switch were not explicitly questioned, in clinical practice, the decision is typically aligned with guidelines, such as the need to improve adherence with a once-weekly dose, further reduce HbA1c, promote a more significant weight reduction, or address secondary cardiovascular prevention (22). Clinicians in developing countries may also need to consider specific situations when deciding to change the molecule, such as the temporary shortage of a particular drug or insurance coverage. Initially, many insurance companies in Colombia reimburse OWS only for patients with cardiovascular disease, potentially explaining the high proportion of patients with a prior myocardial infarction or stroke in our cohort.

Most of the evaluated outcomes, including glycemic control, body composition, and kidney disease, showed positive effects with OWS treatment. The reduction in HbA1c aligns with other reported experiences, demonstrating a decrease of between 1% and 1.5% (16, 19, 20, 23), consistent with findings from the SUSTAIN program (11).

Weight loss is one of the distinctive effects of GLP1-RA use. In the SUSTAIN program with the 0.5-mg dose of OWS, the weight reduction ranges from −3.5 kg (SUSTAIN 4) to −4.6 kg (SUSTAIN 7) and between −4.5 kg (SUSTAIN 1) and −6.5 kg (SUSTAIN 7) with the 1-mg dose. Real-life experiences have been more variable. Some studies report losses of between 3.5 and 5.7 kg (14–16, 19, 23, 24), consistent with our findings. Other studies have reported losses nearing 10 kg (18, 25), likely associated with a longer-term medication use and a high proportion of patients reaching the 1-mg dose. In this cohort, older women experienced a 15% reduction in body weight. Future studies incorporating body composition analysis are necessary to assess whether this substantial weight loss is related to loss of bone or muscle mass (25, 26).

Few observational studies have explored kidney outcomes in patients treated with OWS, and short-term follow-up may be insufficient to detect changes in the eGFR (23, 26). Nevertheless, consistent with some clinical trials (12), a decrease in ACR could serve as an early indicator of renal protection.

Insulin dose reduction is another reported finding in some real-world experiences (27); however, achieving complete insulin withdrawal is not common in patients treated with OWS.

GLP1-RA is notorious for its gastrointestinal adverse events (AEs); however, in our study, the reported AE rate was low, which may be explained by the low report rate of AEs, especially those that are gastrointestinal in nature in clinical practice. Studies that rely solely on EMR tend to underestimate the AE rate; this is a limitation of our study (28).

Our study has other limitations. Firstly, incomplete data for several outcomes, due to the retrospective design, may have affected our estimations. This was compounded by the COVID-19 pandemic, which necessitated virtual follow-ups for several patients and affected the recording of anthropometric measurements such as weight. Although the information originated from different centers, all these institutions provide specialized care, which limits the generalizations of our results. Additionally, information regarding the health coverage of all participants was not collected. In Colombia, OWS is fully reimbursable, but access may differ between the subsidized and contributive regimes; this limits further analysis of the impact of healthcare coverage on OWS effects. Another limitation is that although the mean percentage of weight loss was 5.0%, like other real-life studies, the lack of information regarding the final OWS dose limits our analysis. Finally, the absence of a control group hampers the ability to isolate the effect of OWS.

## Conclusion

The real-life use of OWS in a group of patients in Colombia improves glycemic control and a 5.0% weight loss. Local factors such as guidelines and the availability of other diabetes medications may explain the results. No notable adverse effects were observed during the 1-year follow-up.

## Data availability statement

The datasets produced or analyzed for the present study will be made available upon publication to researchers who provide a methodologically sound proposal. The data will be shared with de-identified individual participant information. Proposals should be directed to the corresponding author, who will contact the sponsor institution (Universidad Pontificia Bolivariana) to obtain permissions. Requests to access the datasets should be directed to DB-C, daisytura88@gmail.com.

## Ethics statement

The studies involving humans were approved by Universidad Pontificia Bolivariana. The studies were conducted in accordance with the local legislation and institutional requirements. Written informed consent for participation was not required from the participants or the participants’ legal guardians/next of kin in accordance with the national legislation and institutional requirements.

## Author contributions

DB-C: Writing – review & editing, Writing – original draft. AG-R: Writing – review & editing, Writing – original draft. CB-V: Writing – review & editing, Writing – original draft. CA-L: Writing – review & editing, Writing – original draft. NC-R: Writing – review & editing, Writing – original draft. CM-A: Writing – review & editing, Writing – original draft. DC-C: Writing – review & editing, Writing – original draft, Methodology, Formal analysis. AR-R: Writing – review & editing, Writing – original draft, Supervision, Funding acquisition, Conceptualization.
