# Supplementary material for: Effectiveness and safety of once-weekly semaglutide: findings from the SEMACOL-REAL retrospective multicentric observational study in Colombia
Source: Front Endocrinol (Lausanne). 2024 Jun 25;15:1372992. doi: 10.3389/fendo.2024.1372992 (PMC11231389; doi:10.3389/fendo.2024.1372992)
Supplement: Supplementary file 1 [file DataSheet_1.docx]

**Supplemenatary material**

**IRB codes and contacts**

Clínica Bolivariana 14-2021 and 15-2022. [comiteetica.salud@upb.edu.co](mailto:comiteetica.salud@upb.edu.co)

Clínica Las Américas-AUNA 190-2022. [cei@lasamericas.com.co](mailto:cei@lasamericas.com.co)

Clínica Medellin 75-2022. [etica.investigacion@imbanaco.com.co](mailto:etica.investigacion@imbanaco.com.co)

Comfenalco Valle, external IRB (Comité de Ética de la Investigación - Riesgo de Fractura S.A) 000002-2022. [comiteetica@cayre.co](mailto:comiteetica@cayre.co)
